# The screening method for use of wild pheasant feathers in the monitoring of environmental pollution with heavy metals

**DOI:** 10.1038/s41598-023-33649-3

**Published:** 2023-04-21

**Authors:** Katarzyna Tajchman, Kamil Drabik, Aleksandra Ukalska-Jaruga, Paweł Janiszewski, Damian Spustek, Karolina Wengerska

**Affiliations:** 1 Department of Animal Ethology and Wildlife Management, Faculty of Animal Sciences and Bioeconomy, University of Life Sciences in Lublin, Akademicka 13, 20-950 Lublin, Poland; 2 Institute of Biological Basis of Animal Production, University of Life Sciences in Lublin, Akademicka 13, 20-950 Lublin, Poland; 3 Department of Soil Science Erosion and Land Protection, Institute of Soil Science and Plant Cultivation, State Research Institute, Czartoryskich 8, 24-100 Puławy, Poland; 4 Department of Fur-Bearing Animal Breeding and Game Management, University of Warmia and Mazury in Olsztyn, Oczapowskiego 2, 10-719 Olsztyn, Poland

**Keywords:** Ecology, Zoology, Environmental sciences

## Abstract

It has been shown that some species of birds, especially herbivorous and territorial ones, are more sensitive to the effects of toxic substances compared to mammals. This allows for taking integrated actions in the area of environmental protection and monitoring in a holistic sense (at various trophic levels). Therefore, this study aimed to assess the possibility of using pheasant feathers (*Phasianus* *colchicus*) as a potential bioindicator ofenvironmental contamination, and thus to determine the concentration of heavy metals (lead—Pb, arsenic—As, cadmium—Cd, chromium—Cr, nickel—Ni, and zinc—Zn) in the analyzed tissue of animals inhabiting the forest districts of the Lubartów, Tomaszów, Skierniewice, and Ostrowiec Świętokrzyski areas. The chemical analysis used to determine the concentration of toxic elements in pheasant feathers was carried out by inductively coupled plasma mass spectrometry. The highest concentrations of Cr and Zn were found in birds from the Lubartów Forest District (1.93 mg/kg and 120.63 mg/kg, respectively), As and Ni in the Tomaszów Forest District (0.55 mg/kg and 1.60 mg/kg, respectively), Cd in the Lubartów Forest District and Skierniewice (0.04 mg/kg), and Pb in the Skierniewice Forest Distict (6.79 mg/kg). The observed results were strongly related to soil contamination and urbanization index, as key environmental factors which significantly determine the metal content in pheasant feathers. Therefore, proposed non-invasive measurements of the elemental composition of feathers of birds living in specific areas may be an important indicator of environmental pollution in relation to the high impact of anthropopressure.

## Introduction

The presence of heavy metals (HMs) in the natural environment is primarily associated with human activity, both in industry and agriculture^1^. HMs affect almost all elements of the ecosystem, from soils^2^ and, aquatic environments^3^, to living organisms. Their harmfulness is related to their transfer from the abiotic environment to living organisms and accumulation in biota at various trophic levels, thus contaminating food chains/webs^4^. The trophic transfer, bioaccumulation, and biomagnification of these substances in food chains have a significant impact on wildlife and human health. HMs are taken through the digestive system bioaccumulate in hard and soft tissues or animal organs and have a long half-life in the body. In addition, they have a high carcinogenic and teratogenic potential and cause kidney or immune system disorders^5–7^; hence, research which that explores their potential for bioaccumulation and biomagnification is very important.

The level of toxicity not only depends on the type of metal but also on the family of animals that are exposed to it. Guitart et al.^8^ showed that birds particularly associated with the aquatic environment or prey are more sensitive to the effects of toxic substances compared to mammals. This sensitivity means that birds are often used in research investigating the content of heavy metals in the environment^9^. This allows us to take integrated actions in the area of environmental protection and monitor it in a holistic sense (at various trophic levels). The ability to accumulate varies in different tissues, but it is assumed that it is higher in organs that show the ability to detoxify, such as the liver, rather than in other tissues of the body^10^. However, it should be noted that obtaining these tissues is impossible in vivo; therefore, alternatives are sought, among which feathers are most often mentioned, as indicated by Kim and Koo^11^ and Malik and Zeb^12^, in the content of heavy metals, especially from sedentary species^13^. In birds, a large proportion of the HMs are found in feathers that are not metabolically active. The metals in these tissues may come from the diet or remobilization of elements from internal tissues^14^. HMs are distributed by various biochemical and physiological processes in the body to the blood, liver, kidneys, and other organs or excreted from the body to feathers, eggs, or feces^15–17^, which can be a mechanism for detoxification^18,19^. In HM feathers, they are associated with sulfur-containing keratin^20,21^, especially mercury, zinc, copper, chromium, arsenic and selenium^22^. For example, a significant concentration correlation has been shown between certain metals (e.g., cadmium) in the blood and feathers of birds of prey^23^. In addition, Dmowski^22^ found that the accumulation of metals in the described tissue, except for mercury, is influenced more by external than internal conditions. The structure of a bird's feather makes them effective particle traps and causes external contamination from solid, liquid, or gaseous sources^24,25^.

Tissue concentrations of HM often increase with bird age^19,26^. Thus, the feathers of young birds and chicks are better suited as indicators of metal contamination than those of older birds^27,28^. Many studies have shown no significant differences in feather metal levels between animal sexes^19,29^. However, in some bird species, concentrations of lead, for example, were much higher in males^30,31^ because females can also excrete lead by oviposition^32^. Local changes in small areas in metal levels in the feathers of raptors may be significant and not adequate^33^, while aquatic birds will rather reflect water pollution^27,34,35^, there are few studies on sedentary species leading a forest and field lifestyle.

In Poland, birds that perfectly reflect the existing environmental conditions due to their strong territorial instinct, and thus attachment to a specific area, include grouse–grouse, black grouse, hazel grouse, partridge, and pheasant^36^. The most common and unprotected species is the pheasant, which prefers small clusters of trees, especially deciduous trees with a dense undergrowth, rather than small forest complexes, stations, and bushes^37,38^. The size of the territory occupied by males is usually less than 5 ha, but it can vary from 0.5 to 45 ha^39^. The food base for adult specimens in winter mainly consists of seeds, berries, small roots, and green parts of plants and grits transforming into gastroliths, which means that the accumulation of harmful substances in its tissues comes mainly from abiotic elements of the natural environment^40,41^. It should also be emphasized that pheasants change their feathers every year after the mating season^39,42,43^ and during this period we can collect them non-invasively, making them an excellent first material to check the current level of accumulation of toxic substances from the last few months.

The natural environment of Poland is characterized by a different degree of pollution depending on the region. Due to the different degrees of anthropogenic pressure (urbanization and share of industry) and natural conditions, individual regions are exposed to environmental pollution at different levels. It is worth emphasizing that the level of heavy metals is usually monitored selectively in agricultural areas, and even less often in agricultural and forest areas where the species in question most often lives; therefore, it is necessary to control their occurrence. For this purpose, a number of methods are used, among which one of the most popular is bioindication. Due to the ability of certain pollutants, such as HM, to accumulate in the tissues of living organisms^44–48^, it is possible to conduct a non-invasive examination by using the feathers of birds^22,49–51^ living in a given area. Therefore, the study aims to assess the possibility of using pheasant feathers (*Phasianus* *colchicus*) as a bioindicator in screening tests of environmental contamination, and thus to determine the concentrations of heavy metals (lead—Pb, arsenic—As, cadmium—Cd, chromium—Cr, nickel—Ni, and zinc—Zn) in the analyzed tissue of animals living in areas with different levels of anthropopressure and industrialization.

## Materials and methods

### Experimental design and sampling

#### Pheasant feathers sampling

**Figure 1:**
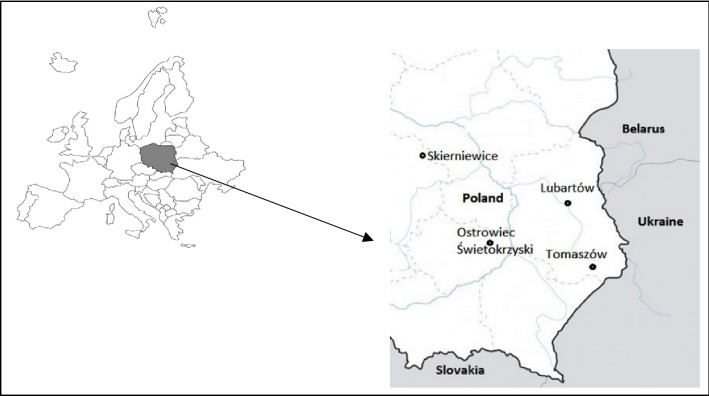
Areas of harvesting pheasants. Generated by the authors using CorelDRAW Graphics Suite 2023 software.

The research material consisted of male pheasant feathers, specifically three remiges of the first and second order (tail feathers), collected immediately after shooting from 64 1–2-year-old pheasants (16 from each area). Such feathers were chosen because they are easy to find and recognize during the natural moults of this species. Pheasants were harvested during the hunting period (February 2022) in accordance with the principles of population and individual selection of game animals in Poland (Polish Hunting Law, Annex to Resolution No. 57/2005 of February 22, 2005) during stalking hunts. The feathers were washed with deionized water and then dried. Immediately before the start of the analyses, the lower part of the quill was cut off from the feathers. The remainder was finely chopped and left for further analysis. Hunting was carried out in four forest districts: the Lubartów Forest District and the Tomaszów Forest District (Lubelskie Voivodeship), constituting potentially uncontaminated areas, as well as the Skierniewice Forest District (Łódzkie Voivodeship) and the Ostrowiec Świętokrzyski Forest District (Świętokrzyskie Voivodeship), constituting areas where urbanization and the industry significantly impact the environment (Table 1, Fig. 1). Selected research areas are recognized in terms of the pheasant population and the condition of the natural environment, and their environmental conditions are representative, which means that they can reflect the level of variable anthropopressure not only in Poland but also in Europe.

**Table 1:** Characteristics of the researched areas^a^.

Forest district	Location	Sum of area—surface (ha)	Afforestation (%)	Arable areas (ha)	Anthropopressure level/population density (people/km)^a^	Urbanization index^a^	Particulates pollutants (t)^a^	Literature
Lubartów	Lubelskie Voivodeship	14,000	23.3	56.3	84	46.38	51,523	https://lubartow.lublin.lasy.gov.pl (access 21.06.2022) Plan Urządzenia Lasu dla Nadleśnictwa Lubartów https://lublin.stat.gov.pl http://eregion.wzp.pl/wskaznik/wskaznik-urbanizacji
Tomaszów		18,690	19.2	66.04				https://tomaszow.lublin.lasy.gov.pl (access 21.06.2022) Plan Urządzenia Lasu dla Nadleśnictwa Tomaszów https://lublin.stat.gov.pl http://eregion.wzp.pl/wskaznik/wskaznik-urbanizacji
Skierniewice	Łódzkie Voivodeship	14,000	15	4.5	136	62.28	3,977,973	Plan Urządzenia Lasu dla Nadleśnictwa Skierniewice https://łódź.stat.gov.pl http://eregion.wzp.pl/wskaznik/wskaznik-urbanizacji
Ostrowiec Świętokrzyski	Świętokrzyskie Voivodeship	17,000	24.3	90	108	45.39	1,528,048	ostrowiec.radom.lasy.gov.pl (access 21.06.2022) Plan Urządzenia Lasu dla Nadleśnictwa Ostrowiec https://ostrowiec.stat.gov.pl http://eregion.wzp.pl/wskaznik/wskaznik-urbanizacji

^a^Data obtained for 2020.

The age of the animals was determined based on length and annual increments on the spur^39^.

All experimental protocols were approved by a University of Life Sciences in Lublin committee. All experiments were performed in accordance with the Polish legislation (Act of 15 January on the protection of animals used for scientific purposes, Journal of Laws 2015 item 266) and the Directive 2010/63/EU of the European Parliament and of the Council of 22 September 2010 on the protection of animals for scientific purposes (Official Journal of the European Union L 276/33) (statement contained in supplementary materials).

#### Heavy metal concentration in soil

 Soil contamination of the reserach area were determined based on the results of The State Environmental Monitoring – The monitoring of the chemistry of arable soils in Poland supervised by the Chief Inspectorate for Environmental Protection. The data has been made available and downloaded from the website https://www.gios.gov.pl/chemizm_gleb/^52^. The results for the researched areas were used in the statistical analyzes of this publication.

#### Heavy metal concentration in pheasant feathers

In order to prepare samples for the analysis of the content of individual elements, the collected feathers were manually crushed and 0.5 g weights were made. The material was not washed before analysis due to fats and waxes on the feathers, which may also be responsible for the sorption of metals from the environemnt (mainly air). The fragmented samples were mineralized with 10 ml of ultrapure HNO_3_ (Merck, Germany) using a MARSExpress microwave mineralizer (CEM, Matthews, NC, USA). In order to prepare samples and blank sample for the AAS and ICP-OES analysis they were ground by using an analytical mill. 0.5 g of the homogeneous mass were weighed by using an analytical balance (with an accuracy of 0.0001 g) and the 10 ml of ultrapure HNO_3_ (Merck, Germany) was added. Mineralization was carried out in teflon vessels (which were cleaned with concentrated HNO_3_ before each digestion) by using a MARS Express microwave mineralizer (CEM, USA). The mineralization was performed stepwise (temperature and power respectively: 90 °C and 400 W, 110 °C and 800 W, 210 °C and 1600 W). After mineralization digestion solution were transferred to 50 ml volumetric flasks and diluted with demineralized water (conductivity 0.055 µS/cm) to the mark. These solutions were used for the further analysis. The commercial certified single-element standard solutions (concentration 1000 mg/l, purity grade—99.999%) were used to prepare calibration curve and supplied by Ultra Scientific. Additionally, during analysis of potassium in order to eliminate sample ionization Schinkel buffer solution (mixture contents 100 g/l lanthanum chloride and 10 g/l cesium chloride) was used.After mineralization, the samples were analyzed using inductively coupled plasma mass spectrometry (ICP OES, Varian Vista MPX CCD ICP-OES equipped with a Vista SPS-5 autosampler) to assess the concentrations of Pb, As, Cd, and Ni. For this purpose, an inductively coupled plasma mass spectrometer 820-MS (Varian, Perth, Australia) was used, while the Zn content, analysis was performed with atomic absorption spectrometry (AAS) using a SpectrAA 280-FS Flame Atomic Absorption Spectrometer (Varian, Australia), as well as an automatic diluent of standards and SIPS tests. Quality control of analytical measurements was performed using home/laboratory reference materials (HRM/LRM) prepared from the feathers of farmed animals living in an uncontaminated environment under controlled conditions. HRM/LRM was used to control the preparation of matrix samples, while standard solutions of metals were used to control the recovery of analytes and the precision of the instruments. In addition, all results were run in duplicate and duplicate samples were used in the series. The results obtained were marked with an accuracy of 5% RSD. The methods used for the analysis were published in earlier works (47).

### Statistical analysis

**Figure 2:**
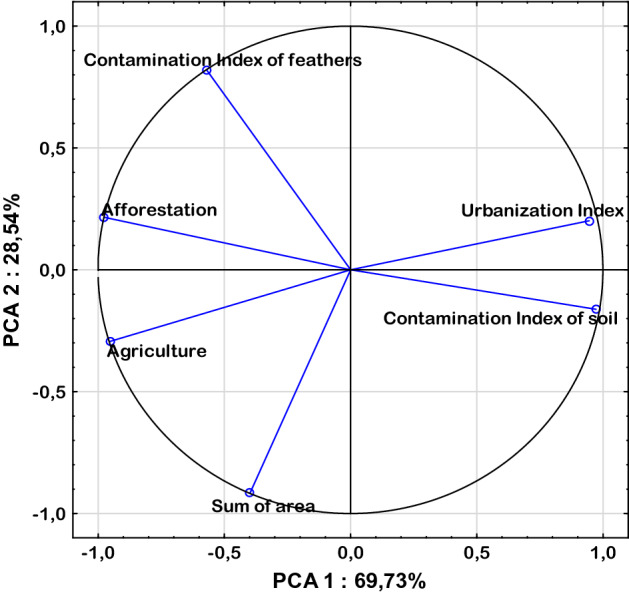
PCA-ordination biplot (component 1 vs component 2) and eigenvectors of correlation matrix used to generate the PCA components of contamination index of feathers and 5 related variables. Arrows/lines in biplot represent variable loadings relative to each component.

The software package SPSS (IBM, 2013) and Statistica (Dell Statistica, version 13.3) was used for statistical analysis. The normality of the data distribution was analyzed with the Shapiro–Wilk test. Differences between the examined districts were determined using a one-way analysis of variance (one-way ANOVA) with Tukey’s multiple-comparison test. The correlations between individual metals were calculated using the r-Pearson correlation. Moreover, principal component analysis (PCA) was used to assess the relationship between variables and the impact of environmental factors on the metal content in pheasant feathers. Differences in metal content in pheasant feather samples were calculated based on the average of individual areas (Tables 2, 4, Fig. 2) while correlations between metal content in feathers were calculated for analyzes performed for each pheasant (Table 3).

**Table 2:** Mean concentration level of heavy metals (mg/kg) in pheasant feathers lived in individual forest districts and of soils^52^.

HM	Lubartów forest district			Tomaszów forest district			Skierniewice forest district			Ostrowiec Świętokrzyski forest district		
	Feathers		Soil	Feathers		Soil	Feathers		Soil	Feathers		Soil
	M	SD		M	SD		M	SD		M	SD	
As	0.14^a^	0.035	3.65	0.55^c^	0.153	4.38	0.30^b^	0.049	1.29	0.21^a^	0.101	3.47
Cd	0.04^b^	0.018	0.50	0.02^a^	0.011	0.50	0.04^b^	0.010	0.50	0.03^ab^	0.007	0.50
Pb	5.83^b^	3.697	10.30	0.92^a^	0.308	10.50	6.79^b^	3.864	9.55	2.97^a^	1.441	54.40
Cr	1.93^c^	0.300	5.63	1.84b^c^	0.299	18.70	1.43^a^	0.191	3.27	1.62^ab^	0.211	13.30
Ni	0.42^a^	0.076	3.46	1.60^c^	0.457	15.10	1.41b^c^	0.666	2.00	1.05^b^	0.272	9.32
Zn	120.63^c^	6.076	22.70	103.74^a^	4.791	29.30	110.81^b^	7.467	4.09	110.88^b^	3.704	33.30

*M* mean, *SD* standard deviation.

^a,b,c^Average differences statistically significant at the level of P < 0.05 in metal content between areas P < 0.05.

**Table 3:** The relationship between heavy metals in the examined tissue of pheasants in total (N = 64).

	As	Cd	Pb	Cr	Ni	Zn
As	1	—0.239	—0.301*	0.023	0.609**	—0.532**
Cd		1	0.241	0.073	—0.284*	0.296*
Pb			1	—0.083	—0.074	0.299*
Cr				1	—0.256*	0.070
Ni					1	—0.540**
Zn						1

*Values statistically significant P < 0.05.

**Values statistically significant P < 0.01.

### Institutional review board statement

Ethical review and approval does not apply for this study, due to pheasants were harvested during the hunting period (February 2022) in accordance with the principles of population and individual selection of game animals in Poland (Polish Hunting Law, Annex to Resolution No. 57/2005 of February 22, 2005).

### Research statements

Reporting in the manuscript follows the recommendations in the ARRIVE guidelines.

## Results

The conducted research showed the diversity in the concentration levels of heavy metals in the feathers of pheasants inhabiting the areas of the analyzed forest districts. The highest contamination rates of Cr and Zn were found in the material from the Lubartów Forest District (1.93 mg/kg and 120.63 mg/kg, respectively), As and Ni in the Tomaszów Forest District (0.55 mg/kg and 1.60 mg/kg, respectively), Cd in the Lubartów Forest District and Skierniewice (0.04 mg/kg), and Pb levels were the highest in the material from the Skierniewice Forest Distict (6.79 mg/kg) (Table 2). Despite slight differences between the content of individual metals in the feathers of pheasants differing in population intensity and industrial emissions, they can be considered an excellent indicator representing the habitat diversity of the research areas. Each area was characterized by different natural conditions; therefore, animals may react differently to metal exposure and ingestion. Nevertheless, although no clear differences were captured in the two groups of areas, the research shows the variability in metal uptake resulting from the living conditions of pheasants.

The Pearson correlation coefficients indicate the interdependencies between the examined elements especially between Zn, Ni, and As (Table 3). It was found that the As concentration level was significantly negatively correlated with the Pb content in pheasant feathers (−0.301, P < 0.05). In the case of the concentration of this element, a highly significant negative correlation with the concentration of Zn (− 0.532, P < 0.01) and a positive correlation with the content of Ni (0.609, P < 0.01) in the analyzed material were also found.

Following an analysis of the interaction between Cd concentration levels and other elements, a significant negative correlation of this metal concentration levels with Ni content was found (−0.284, P < 0.05), as well as a significant positive correlation with Zn (0.296, P < 0.05). Moreover, a significant positive correlation of Pb and Zn (0.299, P < 0.05) was found, while Cr and Ni showed significant negative correlations (−0.256, P < 0.05). It is also worth noting that the content of Ni and Zn in the analyzed samples was highly significantly negatively correlated (−0.540, P < 0.01) (Table 3).

Publicly available data on soil pollution monitoring in Poland were also analyzed (Table 2)^52^. An analysis of the results from our own research and monitoring data showed that certain trends are not confirmed in all the analyzed cases. For example, the concentration levels of As and Ni in both feathers and soil were the highest in the Tomaszów Forest District, and were similarly high but did not reach the highest Cr concentration level in this forest district and Zn in Ostrowiec Świętokrzyski; however, no similar match was found for the other locations (Table 2). This may indicate that different sources of heavy metals had accumulated in the diet of birds.

Further insights into the relationship between the contamination of feathers expressed as the concentration sum of all determined elements (As, Cd, Pb, Cr, Ni, and Zn) and other related variables are shown in Fig. 2 and Table 4. All analyzed data were represented by three main components explaining 100% of the total variance of the results (Table 4), whereas up to 98% of the variance was explained by the first two factors (Fig. 2, Table 4). The first PCA component (component 1) which accounted for 69.73% of the variance was significantly negatively correlated (r > − 0.5) with afforestation and agriculture factors, but was positively correlated (r > 0.5) with contamination and the urbanization index. The second PCA component (component 2), which accounted for 28.54% of the data variance, was significantly positively correlated with the contamination index of feathers and negatively correlated with the sum of the area only. The third PCA factor was not statistically significantly correlated with any of the measured parameters. All of the vectors reached the circuit of the plot; therefore, all variables were well represented by the first two main components of the PCA coordinates. The distance (angle) between vectors confirmed a high mutual impact of all environmental indicators which express the pressure of urbanization and industry on the content of metals in pheasant feathers. The factor loadings matrix (Fig. 2) details the results of the PCA analysis.

**Table 4:** Table of factor loadings matrix, loading ≥ 0.5 are shown in bold.

	Component 1	Component 2	Component 3
Contamination index of feathers^a^	—0.57	**0.82**	0.04
Contamination index of soil^a^	**0.97**	—0.16	0.17
Urbanization index	**0.95**	0.20	—0.26
Sum of area	—0.40	**—0.92**	—0.03
Afforestation	**—0.98**	0.22	—0.01
Agriculture	**—0.95**	—0.29	—0.08
% of variance	69.73	28.54	1.73
Cumulative %	**69.73**	**98.27**	**100**

^a^Contamination index of feathers and contamination index of soil represent the sum of all determined elements (As, Cd, Pb, Cr, Ni, Zn) for individual district.

## Discussion

Our studies show that there are significant differences in the content of individual metals in the feathers of birds from the precincts of individual forest districts. The obtained results in the concentrations of As, Cd, and Zn, despite the differences in the species of the studied birds, show similarity to the data obtained by Grúz et al.^1^, who conducted research on environmental pollution in Hungary using birds of prey. In turn, data from Poland^53^ showed significant differences in the concentration of heavy metals in different tissues of several species of tits. These data confirm our own observations by selecting forest complexes adjacent to regions with varying degrees of pollution. In our research, a comparison of material from birds from habitats surrounded by heterogeneous pollution emitters (transport, industry, and agriculture) was also made. At the same time, it should be noted that the species described by other authors differ significantly from the wild pheasant, if only because of their diet. It has been proven that the diet significantly modifies the content of heavy metals in various tissues of birds^11^. The accumulation of HM in the feathers of birds of prey or those associated with the aquatic environment is very different compared to the animals we studied. These discrepancies may result from the place of individual species in the food chain as well as due to different exposure to the environment in which they lived. The results obtained by Burger^35^ showed in 2011 much lower concentrations of Pb and similar Cd in the feathers of herons nesting in the area of Barnegat Bay (New Jersey), and similar content of As, Cd, Cr, Ni and lower Pb and Zn in *Ardeidae* living in Italy^54^ compared to biological material of pheasants. Larus crassirostris in South Korea showed a similar concentration of As but 4 times higher Cr and Cd and lower Pb and Zn^55^. On the other hand, in the feathers of birds of prey from Finland and Pakistan, concentrations of Cd were two times higher, Pb was very diverse, but also higher, and Zn and Cr were similar^33,56^ to *Phasianus* *colhicus* feathers. The results obtained by Janayedeh et al.^57^ on a house crow in the Klang area of the Malay Peninsula, which was characterized by rapid industrial development, the concentration of Pb was 4–6 times higher, Ni almost 10 times higher, and Zn 2 times higher compared to the researched pheasants.Bearing in mind the above results of the conducted analyses and the fact that species of poking in the soil birds lead a sedentary lifestyle by nature^58,59^, it seems necessary to compare the results of our research with the same family of birds. Data on pheasants are quite modest in this regard. For example, Świergosz^60^ showed significant differences in the level of mercury concentration in the feathers of birds collected in southern and south-eastern Poland. However, other authors analyzing the concentration levels of heavy metals in the tissues of wild turkeys, such as Scanlon et al.^61^, or capercaillies^62^ showed similar, although slightly less significant, relationships regarding the concentration of heavy metals. These differences are probably caused by a large time between their own research and those presented by the mentioned authors, which could have resulted in a significant increase in the content of heavy metals in the environment. Moreover, the difference between metal concentrations in soil and feathers indicates exposure of pheasants to pollutants from anthropogenic sources. Pheasants accumulate pollutants in their feathers (which is associated with an increase in concentration over time), while metals in soils and plants can transform (dynamic processes due to leaching deep into the profile, capture by plants and soil animals,). In addition, pheasants migrate and can inhabit areas with the so-called “hot spot pollution.” An analysis of the relationship between the occurrence of individual heavy metals showed that there are significant interactions between them, which partially coincide with the studies of Szymanowska et al.^63^, which explore the relationship between Cr and Cd. It should be mentioned, however, that these data only indirectly confirm the results of our own research due to the completely different analyzed material. Regardless of the choice of the test material, an analysis of the interdependence between the elements allows, on the one hand, an assessment of the impact of pollutant emitters and their range^64^. On the other hand, this enables an analysis of the environment in terms of its agricultural suitability. It has been shown that some heavy metals interfere with the absorption of ingredients important from the point of view of plant cultivation, contributing to a reduction in the yield^65,66^.

At the same time, it should be noted that the increased concentration levels of metals in the natural environment are primarily caused by human activity. The sources of environmental pollution with heavy metals are industrial emissions (mining, metallurgy, and chemical industries), transport emissions, municipal management (waste storage), and agriculture (use of phosphate fertilizers, lime, and plant protection products). Their subsequent deposition on the surface of plants, soil, or surface runoff can lead to the contamination of entire ecosystems. Toxic elements can enter organisms through the skin and lungs along with polluted air, but the most important impact concerns the alimentary tract through the intake of contaminated food and water^67–69^. Eaten plants absorb toxic substances from the soil; moreover pheasants ingest grit to help digest food in the gizzard. Some of the pollutants found in the feathers of birds can be explained, as in the case of the Skierniewice Forest District, by the proximity of large urban agglomerations, including the capital city. Similarly, for the Ostrów Świętokrzyski Forest District, its location is of great importance. In the Świętokrzyskie Voivodship, there are aggregate mines and cement plants that are a source of dust, the role of which in polluting the natural environment of the smelter has already been quite well known^70,71^, which confirms our thesis that pheasant feathers are a very good bioindicator of environmental pollution.

In the case of the other two forest districts, the role of urbanization is relatively small. At the same time, it should be noted that their locations in agricultural areas have an impact on the content of HMs in the potential diet of pheasants. It has been proven that agricultural production can affect heavy metal pollution^72^. The use of mineral fertilizers (phosphorus and compound) or waste substrates in particular can significantly affect the content of zinc and cadmium in the soil^73,74^.

Other elements, such as arsenic, enter the environment through the burning of fossil fuels, while lead is used to produce gasoline^75^, and therefore is contained in car exhaust fumes deposited on roadside vegetation. It is estimated that the share of atmospheric lead (from dust fallout) in plants may be as high as 73–95%. Cadmium is relatively intensively accumulated by plants, and the source of over 80% of this element are mainly cereals, oilseeds, and vegetables, which are often eaten by pheasants during the growing season.

PCA analysis confirmed the significant impact of environmental factors on the metal content in pheasant feathers. It should therefore be assumed that this type of measurement may be an important indicatior of environmental pollution in relation to the significant impact of anthropopressure. Studies have shown that soil contamination is strongly related to the level of urbanization and negatively correlated with afforestation and agriculture (which had the greatest impacts on the observed correlations of pheasant feather contamination). The observations carried out are additionally valuable because the end of winter is a very difficult period in the life of pheasants as they must quickly rebuild body weight and fat reserves. Thus, foraging during this period is a direct reflection of the birds' exposure to environmental pollution^76^. The obtained results have a significant impact on the development of environmental pollution detection methods in the holistic aspect, i.e. they allow to determine its significant impact on the potential of bioavailability and availability of pollutants. These are very important aspects related to the accumulation of contaminants because their total content does not determine their toxicity. Therefore, the proposed research has a high potential in the field of screening and monitoring methods of environmental pollution by indirectly testing the level of metals in animal organisms. These are also very important issues to limit the direct examination of soils, waters and vegetation that indicate point rather than diffuse contamination. Given the above, the non-invasive analysis of heavy metal content in environments, especially in agricultural or agroforest ecosystems, may indirectly help to reduce the negative effects of heavy metals on the human body, both in terms of the health of the entire population and future generations.

## Conclusions

This first and preliminary analysis of the possible use of wild common pheasant feathers in the monitoring of environmental pollution with heavy metals provided interesting results. Pheasant feathers replaced every year after the mating season are an excellent first material with which to check the current level of heavy metal accumulation over the last few months. In addition, much higher levels of these substances can be expected in other tissues, including game meat consumed by consumers. The conducted research may form the basis for making conclusions about environmental pollution in the analyzed areas, and the obtained results will enable a direct recognition of the main threats related to the proper functioning of agro-forest ecosystems, which are currently quite poorly identified. Moreover, the observed relationships will give hope for the development of this direction of environmental analysis in the aspect of monitoring research.

## Data Availability

All data generated or analyzed during this study are included in this published article.
